# Development of a Middle Cerebral Artery Occlusion Model in the Nonhuman Primate and a Safety Study of I.V. Infusion of Human Mesenchymal Stem Cells

**DOI:** 10.1371/journal.pone.0026577

**Published:** 2011-10-24

**Authors:** Masanori Sasaki, Osamu Honmou, Christine Radtke, Jeffery D. Kocsis

**Affiliations:** 1 Department of Neurology, Yale University School of Medicine, New Haven, Connecticut, United States of America; 2 Center for Neuroscience and Regeneration Research, Veterans Affairs Connecticut Healthcare System, West Haven, Connecticut, United States of America; 3 Department of Neurosurgery, Sapporo Medical University School of Medicine, Sapporo, Japan; 4 Department of Neural Regenerative Medicine, Research Institute for Frontier Medicine, Sapporo Medical University School of Medicine, Sapporo, Japan; 5 Department of Plastic, Hand and Reconstructive Surgery, Hannover Medical School, Hannover, Germany; Universidade de Sao Paulo, Brazil

## Abstract

**Background:**

Most experimental stroke research is carried out in rodents, but given differences between rodents and human, nonhuman primate (NHP) models may provide a valuable tool to study therapeutic interventions. The authors developed a surgical method for transient occlusion of the M1 branch of middle cerebral artery (MCA) in the African green monkey to evaluate safety aspects of intravenous infusion of mesenchymal stem cells (hMSCs) derived from human bone marrow.

**Methods:**

The left Sylvian fissure was exposed by a small fronto-temporal craniotomy. The M1 branch of the MCA was exposed by microsurgical dissection and clipped for 2 to 4 hours. Neurological examinations and magnetic resonance imaging (MRI) were carried out at regular post-operative course. hMSCs were infused 1 hour after reperfusion (clip release) in the 3-hour occlusion model.

**Results:**

During M1 occlusion, two patterns of changes were observed in the lateral hemisphere surface. One pattern (Pattern 1) was darkening of venous blood, small vessel collapse, and blood pooling with no venous return in cortical veins. Animals with these three features had severe and lasting hemiplegia and MRI demonstrated extensive MCA territory infarction. Animals in the second pattern (Pattern 2) displayed darkening of venous blood, small vessel collapse, and reduced but incompletely occluded venous flow and the functional deficit was much less severe and MRI indicated smaller infarction areas in brain. The severe group (Pattern 1) likely had less extensive collateral circulation than the less severe group (Pattern 2) where venous pooling of blood was not observed. The hMSC infused animals showed a trend for greater functional improvement that was not statistically significant in the acute phase and no additive negative effects.

**Conclusions:**

These results indicate inter-animal variability of collateral circulation after complete M1 occlusion and that hMSC infusion is safe in the developed NHP stroke model.

## Introduction

Cellular interventional approaches to improve functional outcome after middle cerebral artery (MCA) occlusion in the rodent have yielded encouraging results. For example, direct or intravenous administration of mesenchymal stem cells (MSCs) and genetically modified MSCs reduce infarction volume and improve functional outcome after cerebral ischemia induction in the rodent [1]–[17]. While these preclinical studies suggest the potential of cell-based therapies to improve outcome in clinical stroke studies, direct extrapolation from the rodent to human cannot be made given issues of species differences and scale. Ideally, demonstration of efficacy in a nonhuman primate (NHP) stroke model would engender greater confidence in the approach since the organization of the vascular and sensory and motor systems in the brain are more similar amongst primates [18]. However, a limited number of stroke studies have been carried out in the NHP using a relatively small number of animals [19]–[23] and the natural course of stroke in the NHP has not been fully studied. In these various models of MCA stroke induction in the NHP there is considerable variation in both lesion size and functional outcome. These differences have been attributed to variation in collateral circulation between NHPs [21] similar to that which can occur in humans.

In this study, we first describe a NHP stroke model where the left M1 segment of the MCA was completely occluded with an aneurysm clip for several hours in the African green monkey (n = 11). The animals were given regular neurological examinations and MRI scans before and during the post-infarction period. Two groups of animals were observed in terms of outcome; severe long lasting hemiplegic (Pattern 1) and modest initial hemiplegic with near complete recovery (Pattern 2). The second objective of the study was to determine the safety of intravenous infusion of a well-defined population of human MSCs (hMSCs) in the acute phase of stroke in the NHP (n = 11). Outcome was evaluated with neurological examinations and MRI scans. There were no adverse events in the hMSC treated group suggesting safety of the cell infusion protocol.

## Results

### Initial series of transient MCA occlusion model in the NHP

**Figure 1:**
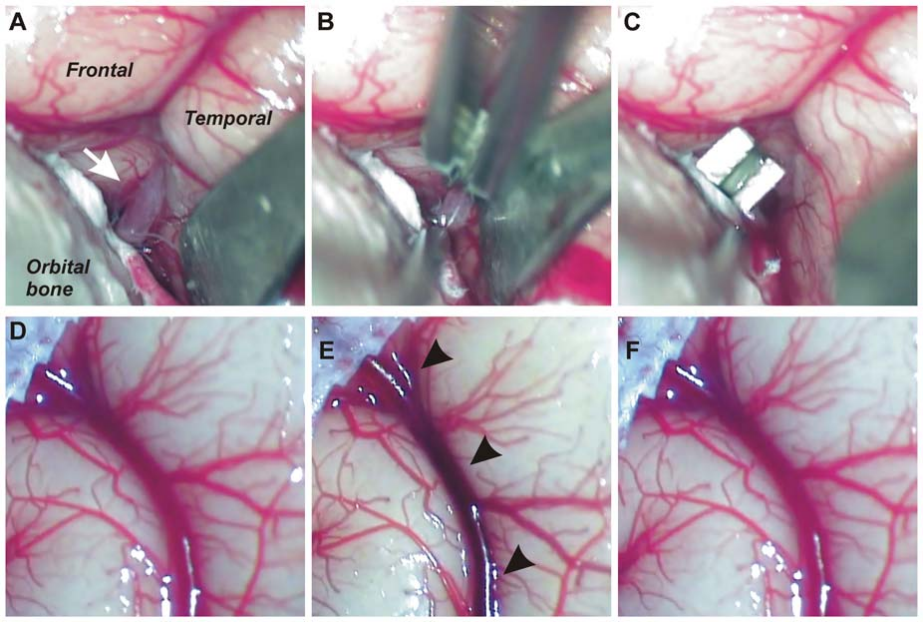
Clipping of M1 portion of left middle cerebral artery with small vascular clip. (**A**) The M1 region **(arrow)** of the MCA was exposed by a retractor deep in the Sylvian fissure. *Frontal* is frontal lobe, *Temporal* is temporal lobe. (**B**) A small vascular clip was applied to the M1 portion. (**C**) MCA was occluded by a clip. (**D**) Before clipping, the color of venous blood was normal. (**E**) Darkning of cortical veins was observed during occlusion of MCA. **(arrowheads)**. (**F**) After releasing the clip, the color of venous blood returned to normal.

In the initial series of experiments to evaluate outcome of the cerebral infarction model in the African green monkey (n = 11) (**Table 1** **,** **2**), a craniotomy was performed to expose the left Sylvian fissure (See Methods and **Figure 1**). The M1 branch of the MCA was exposed in the Sylvian fissure (**Figure 1A**) and was occluded with a vascular microclip (**Figure 1B, C**; n = 10). One animal (#9) had craniotomy alone with no M1 clip for control. Immediately after clipping the M1 portion of the MCA, the vasculature over the lateral margin of the fronto-parietal region darkened (**Figure 1E** **; arrowheads**), small vessels collapsed and a variable degree of reduced cortical blood was observed. In four of the ten NHPs in this set of experiments, venous blood flow was virtually completely blocked. A back and forth movement of a pool of blood within the vessel was observed with vascular brain pulsation, but did not flow to the superior sagittal sinus (Pattern 1). In six of the 10 infarcted animals clipping of the M1 resulted in darkening of cortical blood supply, small vessel collapse, but a slow flow of reduced venous return was observed (Pattern 2). Removal of the clip was followed by return of appropriate coloration of the blood in the cerebral vessels, small vessel reperfusion and return of general blood flow (**Figure 1F**). The difference in venous return is likely the result of animals with rich and with poor collateral circulation. Throughout the study, animals with no observed venous return (Pattern 1) are referred to as “collateral-poor” and those with reduced but observable venous return (Pattern 2) as “collateral-rich” based on this observation during surgery.

**Table 1:** Developing model summary (chronological order).

Animals	Sex	Age	Body weight	MCAO Duration	Collateral	End point
1	F	Unk	4.6	2 hours	Poor	19 M
2	F	Unk	4.5	2 hours	Poor	18 M
3	F	2	5.1	2 hours	rich	3 M
4	F	Unk	5.9	2 hours	Poor	3 days
5	F	Unk	4.6	2 hours	rich	1 M
6	F	11	5.3	2 hours	Poor	13 M
7	F	8	4.2	2 hours	rich	2 weeks
8	F	9	5.5	2 hours	rich	10 days
9	F	8	5	No	craniotomy	4 M
10	F	Unk	4.9	4 hours	rich	2 weeks
11	M	8	6.9	2 hours	rich	2 M

Unk = unknown.

**Table 2:** Developing model parameters.

Animals	HR pre	MABP pre	BT pre	SaO2 pre	HR	MABP	BT	SaO2	HR post	MABP post	BT post	SaO2 post
1	107	55	96.8	100	108	N/A	97.6	100	105	51	98	100
2	110	52	97	100	112	57	96.3	94	117	56	99	99
3	115	78	95	98	118	81	95.4	98	112	82	96.5	99
4	114	68	96	100	121	60	97	100	116	56	97	100
5	116	54	97	99	120	55	97.2	99	132	55	96.8	100
6	150	60	99	99	156	56	98	99	147	59	98	100
7	139	59	97	99	134	60	98.7	100	135	52	98	99
8	142	54	100	98	145	57	101	97	147	58	99	100
9	126	55	96	99	118	58	95.6	100	115	57	97	99
10	115	56	97	99	120	58	94.4	99	128	54	97	99
11	103	64	97	99	95	66	94.1	98	98	63	96	98

HR = heart rate; MABP = mean arterial blood pressure; BT = body temparature; SaO2 = oxygen saturation.

**Figure 2:**
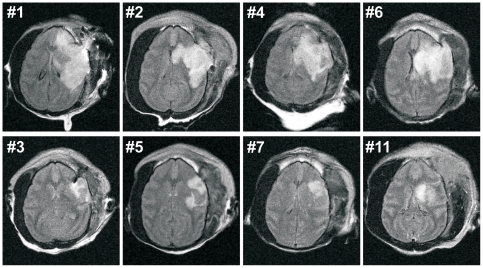
MRI of model development (initial) series. Axial images of MRI FLAIR at day 3 obtained from all animals in the model development (initial) series. Upper panels (#1, #2, #4, #6) are collateral-poor animals, lower panels (#3, #5, #7, #11) are collateral-rich animals. High intensity area indicating cerebral infarction is primarily located in the left basal ganglia in collateral-rich animals. There was more extensive infarction areas in the MCA territory including cortex in collateral-poor animals.

**Figure 3:**
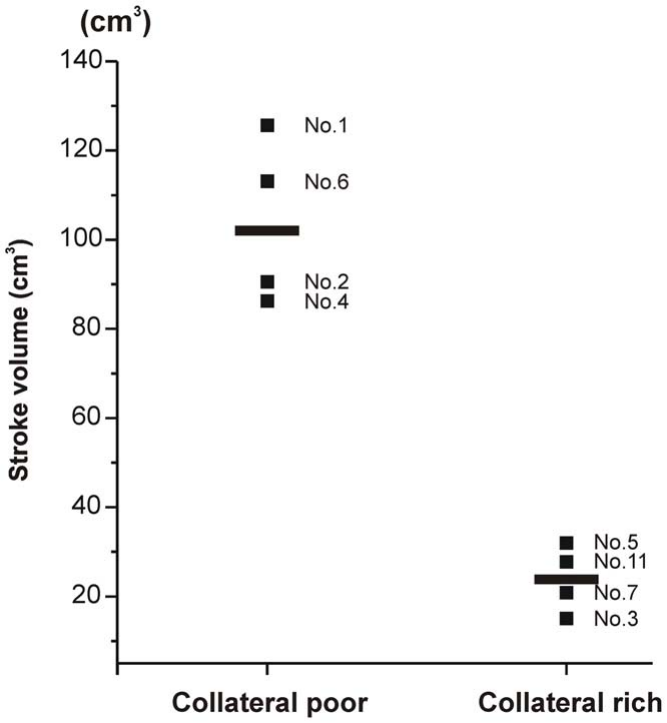
Scattered plots of stroke volume from model development series (n = 4/group). Scattered plots show the stroke volume measured with axial images of MRI FLAIR at day 3. The stroke volume of collateral poor group (103.9±18.7 cm^3^) is greater than the collateral rich group (23.9±7.5 cm^3^; p = 0.0032). Each black symbol with number corresponds to the animal number in Table 1. Long symbols show averages. Given the small size, this was confirmed with a nonparametric test, the Mann-Whitney *U* test (p = 0.029).

**Figure 4:**
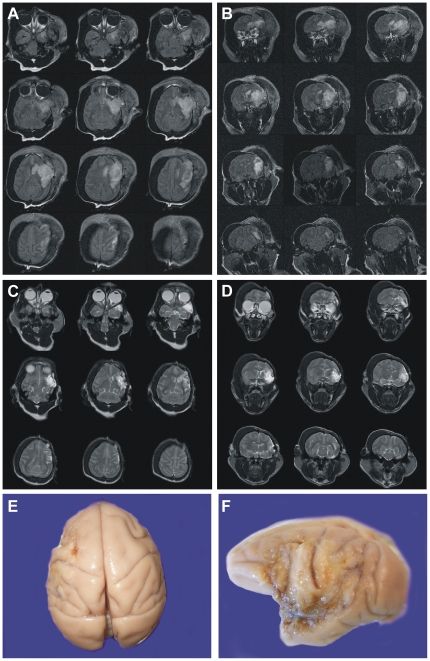
A representative animal of collateral poor NHP (2 hour occlusion of MCA). Axial (**A**) and coronal (**B**) MRI FLAIR at day 3 demonstrate extensive high intensity areas located in the left MCA territory. Axial (**C**) and coronal (**D**) image of MRI T2WI at 1-year post-stroke demonstrated that high intensity areas remained. Gross brain anatomy revealed obvious lesion on the lateral surface of the brain 1 year after stroke (**E, F**).

**Figure 5:**
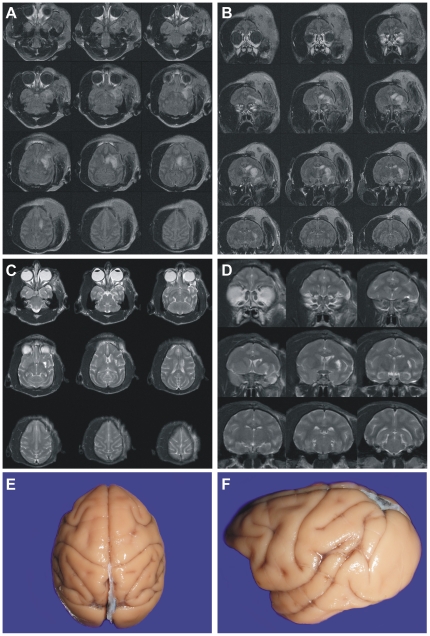
A representative animal of collateral rich NHP (2 hours occlusion of MCA). Axial (**A**) and coronal (**B**) MRI FLAIR at day 3 demonstrate high intensity area located primarily in the left basal ganglia. Axial (**C**) and coronal (**D**) image of MRI T2WI at 1-year post- stroke demonstrated high intensity area is reduced. Gross brain anatomy revealed no obvious lesion in the surface of the brain at 1 year after stroke (**E, F**).

MRI (FLAIR, axial plane) obtained from 2-hour occlusion animals at day three are presented in **Figure 2**. High intensity areas indicating infarcted lesion were localized primarily in the basal ganglia and in the MCA territory including cortex. Stroke volume of each animal demonstrated smaller infarcted areas [**Figure 2** **, lower panels**; 23.9±7.5 cm^3^ (mean ± SEM)] in the collateral-rich group as compared to the collateral-poor group [**Figure 2** **, upper panels**; 103.9±18.7 cm^3^ (mean ± SEM); p = 0.0032, *t* test]. There was a 23% decrease in the collateral-rich group. This was confirmed with a nonparametric test, the Mann-Whitney *U* test (p = 0.029). MRI could not be performed from an animal with 2-hour occlusion (#8) because of motion artifact. No infarcted areas were observed in the animal with craniotomy alone (#9). These data are summarized graphically in **Figure 3**. Examination of the gross brain of a representative animal in the collateral-poor group indicated severe necrosis of the left MCA territory at 1 year (**Figure 4E, F**), but virtually no gross changes could be observed in the collateral-rich animals (**Figure 5E, F**). In one NHP (#10), we increased the occlusion time to four hours from two hours and stroke volume at day 3 was 43.97 cm^3^. Although this animal showed a cortical circulation pattern during and after occlusion similar to the collateral-rich group described above, the general health status of this animal was very poor as compared to other 2-hour occlusion animals, and the Yale Veterinary Clinical Services (VCS) recommended to euthanize after 2 weeks of stroke induction.

### Effects of hMSC infusion of animal health, stroke volume and motor performance

After completion of the initial series to assess the lesion model, hMSCs or human serum was infused into the animals 1 hour after clip release (n = 11). Both groups were immunosupressed with Cyclosporin A (See Methods). Occlusion time was 3 hours in the safety study for both the cell infusion and human serum groups (**Tables 3** **,** **4**) because previous studies using *Macaca* demonstrated that 3 hours of occlusion is sufficient to cause a permanent stroke in the NHP [21], [24]. Ten animals with collateral-rich circulation were used for the analysis to compare the neurological scores and stroke volume. To evaluate whether hMSC intravenous infusion produced adverse effects, we assessed the general vital signs of each animal prior to surgery and monitored them after surgery on a regular basis. No apparent changes in general physical examination were evident in any hMSC infused animals during the survival period except the expected right motor deficits that either did not worsen or improved, which was qualitatively similar to control. It should be noted that one animal (#21) in the serum group died at day 3 due to an unknown reason and another animal (#13) in the serum group became comatose and had to be euthanized at 7 days post-surgery. Autopsies were obtained by Yale VCS group and showed no obvious medical complications in their organs, but they displayed massive hemorrhagic infarction.

**Table 3:** Safety Study animals summary.

Animals	Sex	Age	Body weight	MCAO Duration	Collateral	Treatment	End point
12	M	Unk	5.7	3 hours	rich	Cells	12months
13	F	8	5.5	3 hours	rich	Serum	7 days
14	F	Unk	4.1	3 hours	rich	Cells	12months
15	F	12	5.6	3 hours	rich	Serum	12months
16	M	Unk	6.2	3 hours	poor	Cells	12months
17	M	Unk	5.5	3 hours	rich	Cells	12months
18	M	Unk	5.3	3 hours	rich	Cells	12months
19	F	Unk	4.9	3 hours	rich	Serum	12months
20	F	Unk	4.1	3 hours	rich	Serum	12months
21	F	Unk	4.5	3 hours	rich	Serum	4 days
22	F	Unk	5.2	3 hours	rich	Serum	12months

Unk = unknown.

**Table 4:** Safety Study animals parameters.

Animals	HR pre	MABP pre	BT pre	SaO2 pre	HR	MABP	BT	SaO2	HR post	MABP post	BT post	SaO2 post
12	119	52	96.3	99	125	48	96.3	99	126	51	97.7	99
13	120	50	96	99	122	52	95	95	124	54	97	100
14	130	53	95.4	98	129	47	97.1	99	133	55	98	99
15	134	45	98.2	100	152	51	100	98	138	49	97	100
16	124	49	97.6	100	110	56	98.4	100	118	53	97	99
17	139	55	96.5	97	139	52	101.4	99	136	54	97	99
18	112	52	98.5	98	118	52	98.7	98	115	53	98	100
19	140	58	98	99	145	53	98.9	99	143	48	96.8	100
20	128	59	99	100	123	56	99	97	129	58	97.8	100
21	162	57	98.5	99	165	58	98.7	95	150	54	96.8	97
22	135	69	98	100	132	72	99.4	99	130	65	96.9	98

HR = heart rate; MABP = mean arterial blood pressure; BT = body temparature; SaO2 = oxygen saturation.

**Figure 6:**
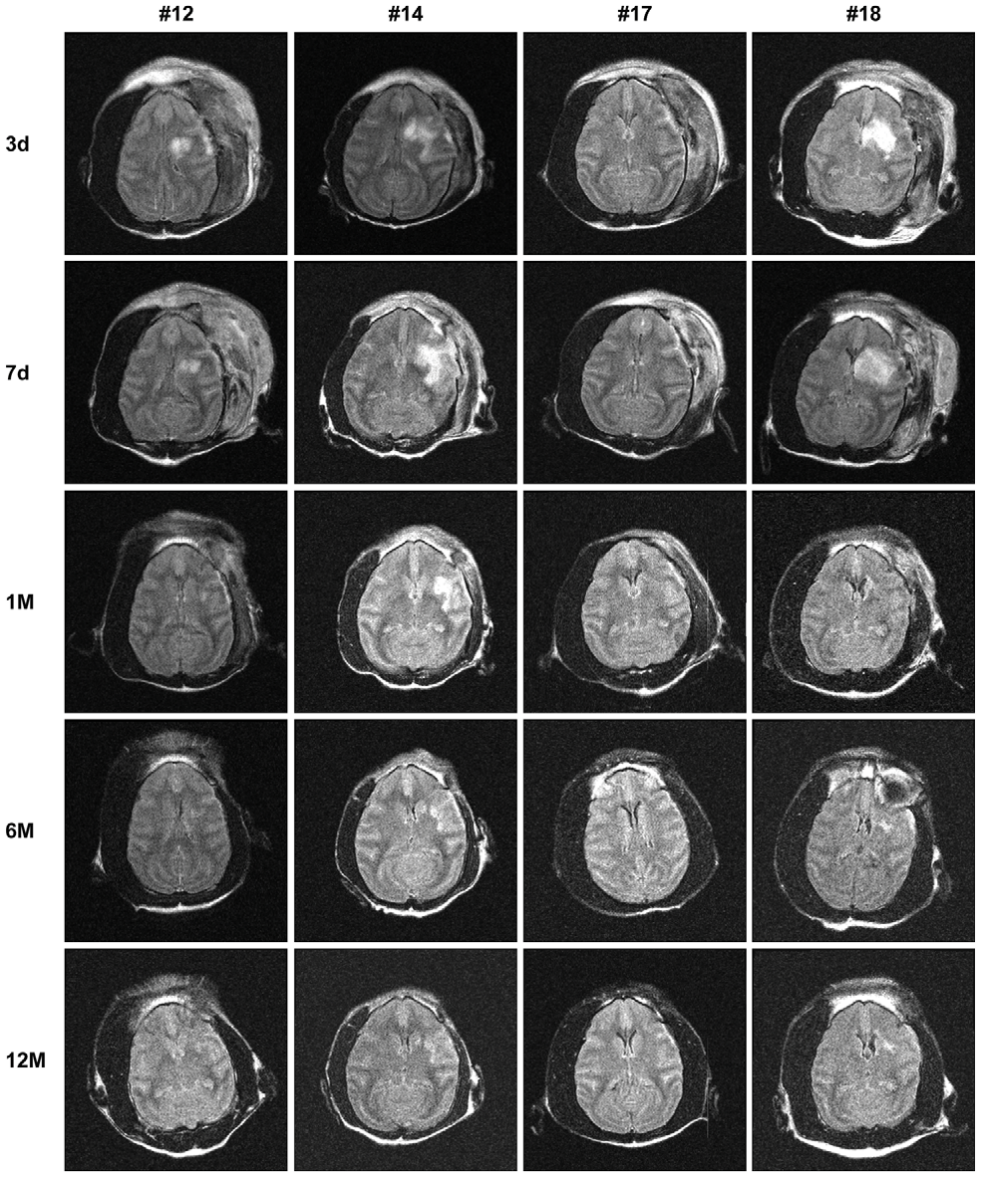
MRI FLAIR images of hMSC infused NHPs. Axial images of available MRI of hMSC infused NHPs at day 3, day 7, 1 month, 6 months and 12 months. #12, #14, #17 and #18 correspond to the animal number in Table 3.

MRIs from all NHPs with collateral-rich at 3 days, 7 days, 1 month, 5–6 months and 12 months after surgery with serum or hMSC infusions are shown in **Figure 6** **and** **7** respectively. As presented in **Figure 8**, the stroke volumes of hMSC infusion group were smaller than the serum infusion group at day 3 (101±39.13 cm^3^; 70.14±23.63 cm^3^; p = 0.54, *t* test), day 7 (80.8±35.66 cm^3^; 61.27±22.37 cm^3^; day 7; p = 0.65, *t* test), 1 month (41.63±25.79 cm^3^; 24.44±15.99 cm^3^; p = 0.59, *t* test), 6 months (24.63±17.95 cm^3^; 16.78±13.84 cm^3^; p = 0.74, *t* test) and 12 months (20.02±15.73 cm^3^; 15.85±14.13 cm^3^; p = 0.84, *t* test). Repeated Measures ANOVA indicated no interaction between the two groups. Although a trend was observed, comparison of the stroke volume between hMSC and serum infusion groups at all time points did not reach statistical significance. This was confirmed with the Mann-Whitney *U* test.

**Figure 7:**
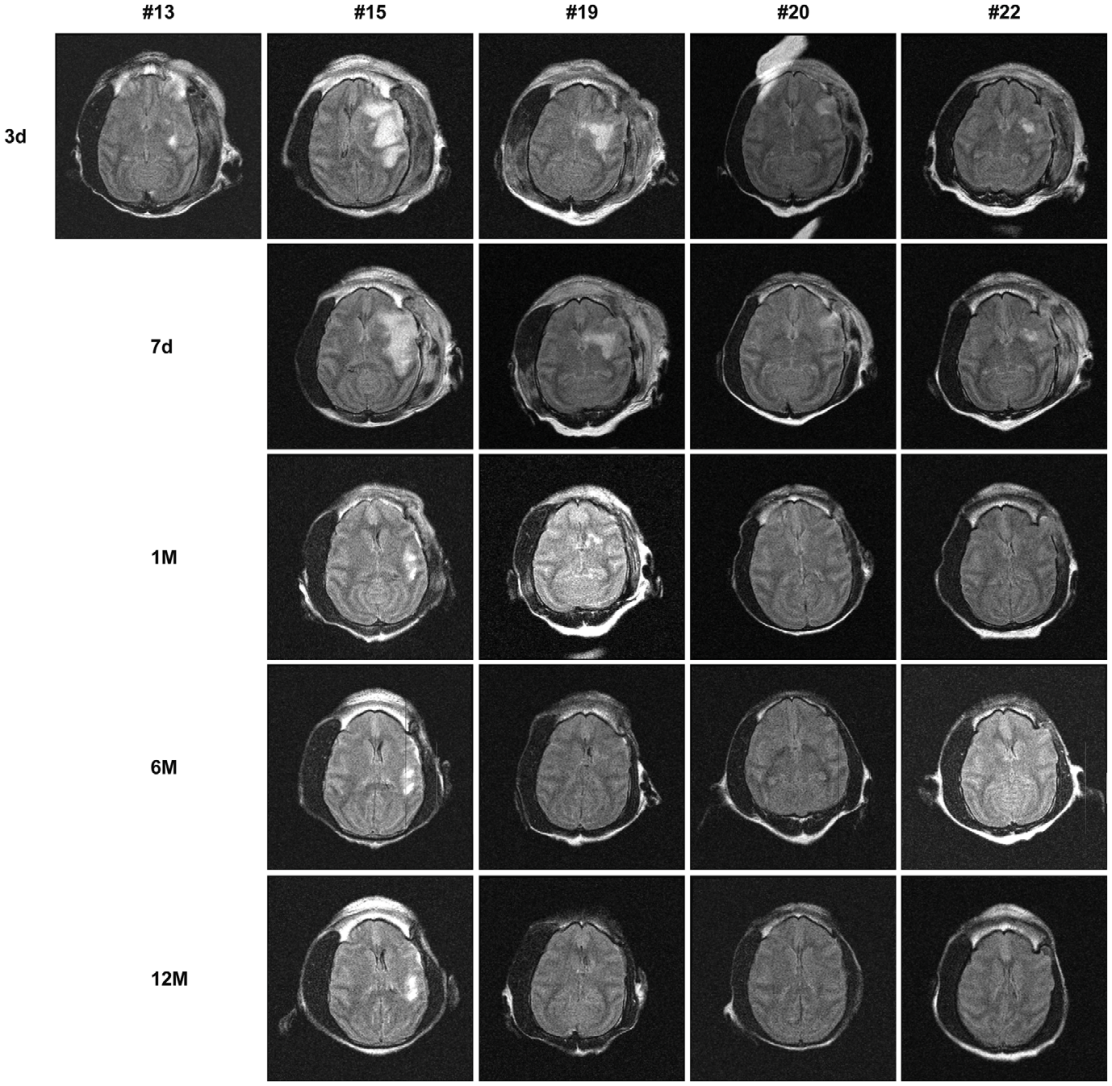
MRI FLAIR images of serum infused NHPs. Axial images of available MRI of serum infused NHPs at day 3, day 7, 1 month, 6 months and 12 months. #13, #15, #19, #20 and #22 correspond to the animal number in Table 3. #13 died at day 7.

**Figure 8:**
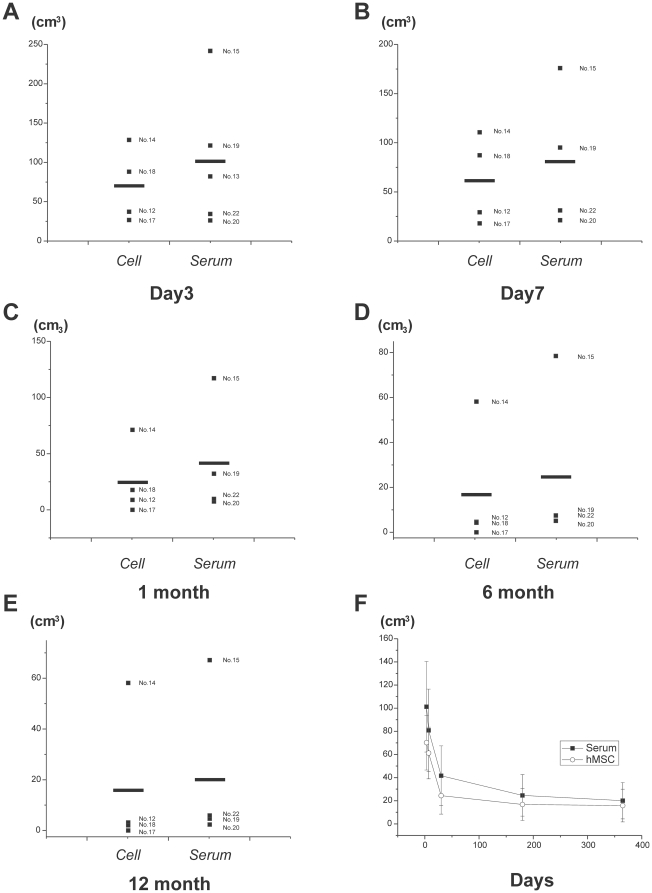
Summary of stroke volume from the safety study group. Measured stroke volume (day 3, day 7, 1 month, 6 months and 12 months) in the hMSC and serum infused animals (**A–E**). Each small symbol with number represents a single animal. Long symbols are averages. Across all MRI methods, there was an overall decrease in stroke volume, but no statistical significant difference. Summarized time-course is shown in **F**.

**Figure 9:**
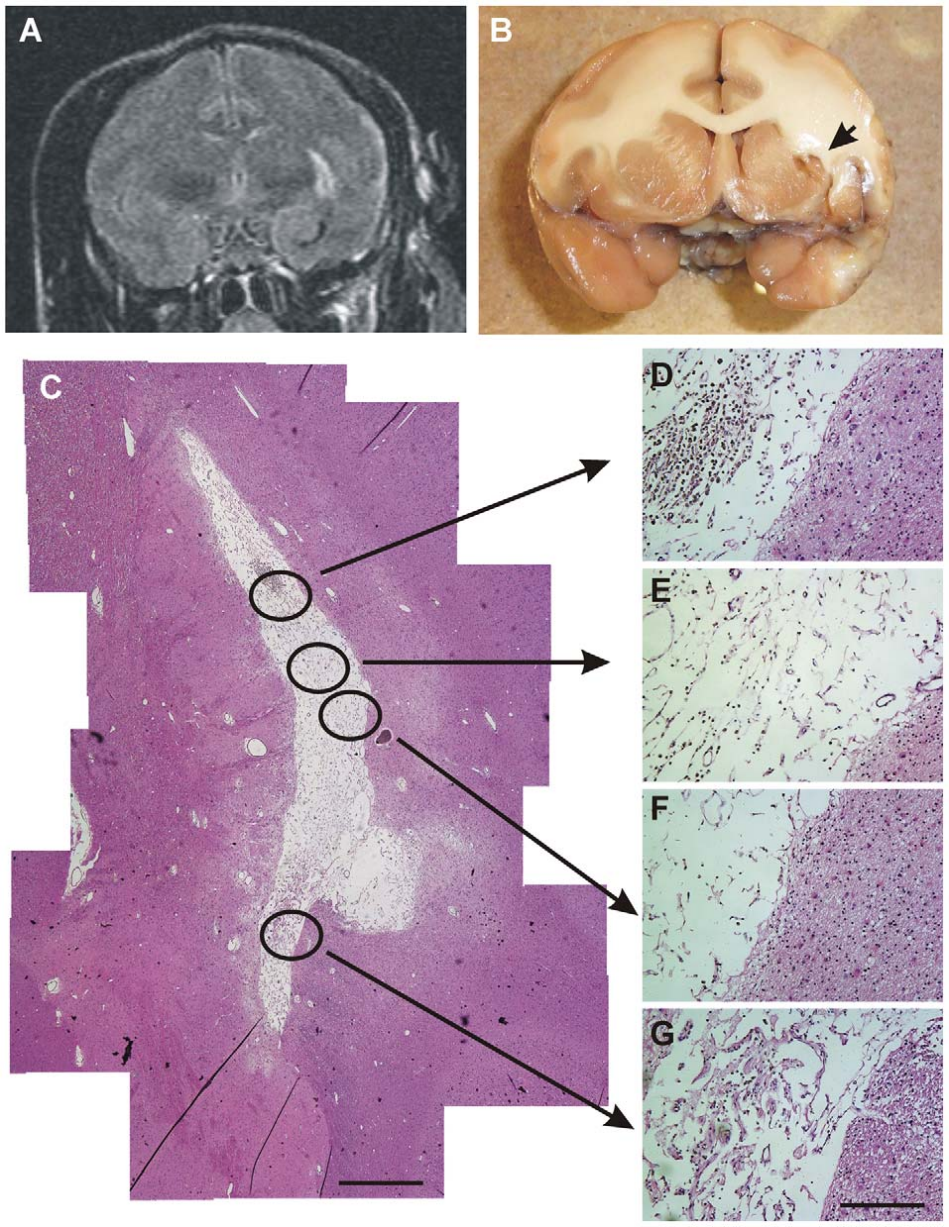
A representative hMSC infused NHP (#14). (**A**) Coronal image of MRI FLAIR obtained at 12 months after infusion of hMSCs demonstrate high intensity area indicating stroke in the left basal ganglia and macroscopic image (**B**) shows necrotic cavity **(arrow)**. Montage image (**C**) of HE staining of frozen coronal sections revealed cavity formation in the basal ganglia and no tumor formation. High power images of HE staining revealed infiltration of inflammatory cells in the cavity and fibrous gliosis in the wall of the cavity. No apparent infused hMSCs can be found in the lesion area. Scale bars = 1 mm (**C**), 200 µm (**D–G**).

MRI (**Figure 9A**) and a macroscopic image (**Figure 9B**) obtained from a representative animal (#14) in the hMSC infused group demonstrated small necrotic cavity in the left basal ganglia. HE staining (**Figure 9C–G**) from a same animal revealed infiltration of the inflammatory cells in the formed cavity, fibrous gliosis in the wall of the cavity and no tumor formation in and around the cavity, even after one year. Obvious survival of the hMSCs was not observed with anti-human nuclei staining (data not shown).

**Figure 10:**
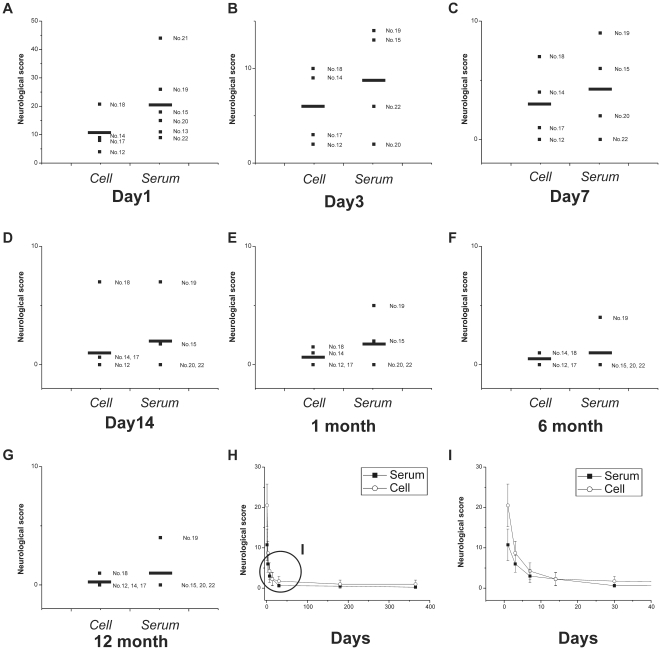
Neurological scores. Neurological score in the hMSC and serum infused animals at day 1 (**A**), day 3 (**B**), day 7 (**C**), day 14 (**D**), 1 month (**E**), 6 months (**F**) and 12 months (**G**). Each small symbol with number represents a single animal. Long symbols indicate averages. There was an overall significant decrease in Neurological score in hMSC group, but no statistical significant difference between the groups at all time points. Summarized time-course is shown in **H, I**. Circled area in H is expanded in (**I**).

Neurological scores for hMSC and serum infusion groups with collateral rich animals were tested at day 1, day 3, day 7, day 14, 1 month, 6 months and 12 months after surgery (**Figure 10**). All experimental animals exhibited a gradual improvement in Neurological score. Statistical analysis indicated that the Neurological scores of the hMSC infusion group were lower than the serum group at day 1 (10.75±7.80; 20.5±12.97; p = 0.219, *t* test), day 3 (6.0±4.08; 8.75±5.73; p = 0.464, *t* test), day 7 (3.0±3.16; 4.25±4.03; p = 0.642, *t* test), day 14 (2.25±3.20; 2.25±3.30; p = 1, *t* test), day 30 (0.625±0.75; 1.75±2.36; p = 0.3399, *t* test), 6 months (0.5±0.57; 1.0±2; p = 0.647, *t* test) and 12 months (0.25±0.5; 1.0±2; p = 0.494, *t* test) after surgery respectively. Although a trend was observed, these differences did not reach statistical significance. This was confirmed with the Mann-Whitney *U* test. Repeated Measures ANOVA indicated no interaction between the two groups.

## Discussion

We first describe a stroke model in the African green monkey (old world monkey) that can be induced by a microsurgical procedure via open supratentorial craniotomy and microclip occlusion of the M1 portion of the MCA. Two general groups of animals were observed in terms of functional outcome and ischemic volume. One group (Pattern 1) displayed severe hemiparesis, and MRI and gross brain examination demonstrated more extensive cerebral infarction in the MCA territory including cortex. The second group (Pattern 2) displayed minor hemiparesis, and MRI exhibited a localized infarcted lesion primarily in the basal ganglia. Direct visualization of the blood “pooling” in cortical veins during M1 clipping provided a clear indication of the collaterality in the brain. In the more severe lesions (Pattern 1), venous return was blocked during clipping, but in the less severe lesions (Pattern 2) venous return was greatly slowed, but could be visualized by microscopic examination of surface veins during surgery. This suggests that the degree of collateral circulation varied in the animals, with the most severe lesions being in animals with poor collateral circulation. We thus grouped the animals as collateral-rich and collateral-poor based on this observation during surgery. The differences between the two groups are not likely the result of incomplete occlusion by the clip. In one NHP where collateral rich circulation was observed after M1 clipping, the animal was immediately euthanized. Observation of the M1 clip at the clip site in the Sylvian fissure revealed a complete occlusion of the vessel indicating incomplete clipping was not the reason for lesion variability. Thus, variance in collateral circulation between the NHPs likely explains the differences in lesion severity. We tried to measure cerebral blood flow with laser doppler flowmetry (Laser Doppler ALF21, ADVANCE CO., LTD., Tokyo, Japan) in some animals, however, the laser doppler flowmetry provided information on the relative decrease in blood flow at only a single point, thereby making it difficult to evaluate the collateral circulation in the African green monkey's brain and determine whether the territory is fully or only partially occluded as described in a canine stroke model [25]. Future studies with laser Doppler scanner or advanced MRI imaging will be required. We would stress that there are limitations on extracting conclusions from the model development (initial) series. In this study, we used eleven animals for model development series and found two patterns of changes. Although eleven NHPs are relatively large number compared to the previous NHP stroke studies [19]–[23], there were budgetary and ethical issues to use NHP compared to rodent studies in general. Thus we were not able to increase the number of experimental animals to provide consistent lesion volumes which are observed in the rat MCA occlusion model [26].

To help address barriers in the translation of animal studies to human clinical trials, the Stroke Therapy Academic Industry Roundtable (STAIR) publication provided recommendations for the preclinical development of acute ischemic therapies [27]. The STAIR reported in their initial publication that there are no standardized, well-accepted models of stroke recovery in primates, although limited experience exists with baboons [18]. A panel of investigators in spinal cord injury research commented that studies in NHP can probe the effects of therapy-induced neural plasticity on multiple aspects of functional recovery with a refinement that cannot be attained in rodents and recommended the use old world monkeys [28]. One reason to use old world monkeys is that they have the advantage of being easily trained to assess function, and the projection patterns of its corticospinal tract (including direct connections with motoneurons) and its nonprimary motor cortical areas are more similar to those of humans.

Over the past 10 years, in addition to the microsurgical approach via open craniotomy or transorbital approaches [29], [30], photochemical [19], [23], and chemical [20] approaches have been developed in NHPs. Several stroke models in medium-sized animals such as pigs [31], sheep [32] and canines [25] also have been developed. Recent advances in interventional neuroendovascular technique have made possible the establishment of a stroke model in macaques [21]. An advantage of this endovascular technique is that it is less invasive compared to our surgical approach, but there are still some complications, such as potential arterial dissection [33] or incomplete occlusion. In rodents, the intraluminal thread model of MCAO is widely used and produced with blind insertion of the microsuture to occlude the origin of the MCA [26], [34]. However, to carry out a similar technique in a NHP, a fully equipped neuroradiological facility with a highly skilled endovascular neurosurgeon utilizing a fine microcathether for intraluminal occlusion would be needed [21].

While we and others [29] have employed a supratentorial craniotomy approach which adds an additional potential surgical risk as opposed to an endovascular approach, clinical experiences indicate that it is relatively safe. Only 0.8% of 4,992 patients who underwent intracranial procedures experienced postoperative hemorrhage [35], and we observed no major complications that could be attributed to the craniotomy. While the craniotomy appears to pose low risk in the direct clip model, variation in collateral circulation in the NHP for any model thus far described leads to variability in outcome between animals complicating comparison between treated and non-treated groups, despite presenting a model more similar to stroke in humans. Indeed, de Crespigny et al. (2005) who used the endovascular approach with 3 hours occlusion in macaques also attribute the relatively good outcome, despite angiographically confirmed occlusion by microcatheter tip, as being due to good collateral circulation [21].

Intravenous infusion of autologous hMSCs is a potentially promising approach for stroke therapy. Rodent work suggests reduction of stroke volume and functional recovery is induced after intravenous infusion of hMSCs [1]–[3], [6], [8], [11], [12], [15], [16]. Current thinking is that the potential beneficial effect of MSCs in various experimental models of CNS injury is not from neuronal or glial differentiation but from release of trophic factors which may provide for neuroprotection [36]–[38], induction of axonal sprouting [39], neovascularization [12], and immunomodulation [40], [41]. In rodent studies, infusion of hMSCs genetically modified to produce various trophic and angiogenic factors leads to reduction in lesion volume and improved functional outcome [3], [8], [12], [42]–[45]. Several institutions including us have carried out Phase I clinical trials with intravenous autologous bone marrow transplantation for stroke patients and have reported preliminary results [46]–[49].

Phinney and colleagues performed safety studies of direct hMSC injection in the right caudate nucleus of healthy adult rhesus macaques and reported no adverse effects [50], [51]. We studied the safety of intravenous infusion of hMSCs in NHPs after stroke induction. The cells were delivered in the acute phase at 1 hour after clip release. No adverse effects were observed in the hMSC group as compared to animals infused with human serum alone. Importantly, MRI and histological examination of the infarction site indicated no tumor formation. HE staining revealed infiltration of macrophages in the formed cavity and fibrous gliosis in the wall of the cavity, however, survival of the hMSCs was not identified with anti-human nuclei staining. Both the serum and the hMSC groups showed functional recovery and tendency to reduce in infarction volume over time. Differences of infarct volume evaluated by MRI and neurological improvement observed in the hMSC and serum groups did not reach statistical significance, but a trend was observed. It should be noted that neuroprotective effects by cyclosporine may participate in the reduction of stroke volume and functional improvements in both groups. Although it is not clear the reason that no animals died in the initial model development group but two animals with human serum infusion had severe hemorrhagic complications lead to death within 7 days in the safety study group, it should be noted that the time of purchase for the animals were different between two experimental series. It is possible that there were unknown differences of environmental, genetical and growth history.

In summary, this study shows that surgical occlusion (clipping) of the M1 region of the MCA for several hours results in two classes of lesion severity; mild and severe. The mild lesion animals have greater collateral circulation than the severe group. MRI analysis indicated that the majority of the animals with MCA occlusion displayed mild neurological symptoms and relatively small infarction sites in deep structures including the basal ganglia with little cortex involvement. The severe group had extensive contralateral hemiplegia and necrosis in the MCA territory. Future studies will be required to predict the collateral circulation quantitatively using intra-operative electroencephalography and/or somato-sensory evoked potential recording in addition to the direct visualization of the blood “pooling” in cortical veins during M1 clipping reported in this study. In addition to the predicting the collateral circulation, the measurement of brain tissue reorganization with perfusion-weighted MRI and/or diffusion tensor MRI might be useful to understand vascular and neuronal remodeling after stroke in NHP. Comparison of neurological function and lesion volume between animals infused with hMSCs or human serum without cells indicated no statistical difference in outcome although a trend was observed. The variability in functional outcome in this and other studies in NHP is likely the result of variable collateral circulation. This variability is certainly a complicating factor for studies designed to assess efficacy. However, with regard to safety, there were no adverse events observed in the NHPs infused with hMSCs. Additional studies will be necessary to increase the number of animals to determine if the trends observed in lesion volume reduction and improved neurological scores in the hMSC group may reach significance.

## Materials and Methods

### Cell preparation and characterization

This study was conducted according to the Declaration of Helsinki guidelines and was approved by the Institutional Review Board at Sapporo Medical University. We received written informed consent from all participants. hMSCs were prepared by using a previously described method with minor modifications [52]. Briefly, peripheral blood (200 ml) for human serum and bone marrow (30 ml) were collected from healthy volunteers. Bone marrow was obtained from the posterior iliac crest under local anesthesia, and was diluted with Dulbecco's modified Eagle's medium, (Mediatech Inc, Manassas, Virginia) supplemented with 10% human serum, 2 mM L-glutamine (SIGMA-ALDRICH, St. Louis, Missouri), 100 U/ml penicillin-streptomycin (SIGMA-ALDRICH, St. Louis, Missouri), plated on 150-mm Tissue Culture Dish (IWAKI, Tokyo, Japan), and incubated in a humidified atmosphere of 5% CO_2_ at 37°C for several days. hMSCs were selected by plastic adhesion and nonadherent cells were eliminated by replacing the media. When cultures almost reached confluency, the adherent cells were detached with trypsin–EDTA solution (Mediatech Inc, Manassas, Virginia) and subcultured at 1×10^4^ cells/ml. The hMSCs were expanded and cryopreserved until usage. Cell passages were limited to three or less, targeting a cell number of 1.0×10^8^ cells. Cryopreservation permitted detailed characterization of cells and pathogens, which required several days prior to cell infusion and resulted in higher cell viability (>95.2%).

### Cell characterization and pathogen screening

Flow cytometric analysis of hMSCs was performed as previously described [6], [8]. Briefly, cell suspensions were washed twice with PBS containing 0.1% bovine serum albumin (BSA). For direct assays, aliquots of cells at a concentration of 1×10^6^ cells per milliliter were immunolabeled at 4°C for 30 minutes with the following antihuman antibodies: PE–conjugated CD34, CD45, (Becton Dickinson Bioscience Pharmingen, San Jose, CA) and CD105 (BioLegend, San Diego, CA). As an isotype-matched control, mouse immunoglobulin G1 (Becton Dickinson Bioscience) was used. Labeled cells were analyzed by a FACS Calibur flow cytometer (Becton Dickinson) with the use of FlowJo software. Dead cells were gated out with forward-versus side-scatter window and propidium iodide staining. The expanded hMSCs were tested for sterility; there was no evidence of bacterial, fungal, viral (ATL, HBV, HCV, HIV), or mycoplasmal contamination, and endotoxin level was under non-pathogenic level in all samples.

### *Animals*

All animal experiments were performed at the Veterans Affairs Connecticut Healthcare System in strict accordance with National Institutes of Health guidelines for the care and use of laboratory animals and in accordance with the recommendations of the Weatherall Report. The Veterans Affairs Connecticut Healthcare System Institutional Animal Care and Use Committee approved all animal protocols (JK-0015). We also followed the Nonhuman Primate Environmental Enrichment Program by the Veterans Affairs Connecticut Healthcare System. Twenty-two young adult African green monkeys (3.5–5.0 kg) of St. Kitts origin (*Chlorocebus sabaeus*; Vervet; old world monkey) were used. Eleven of 22 animals were used for development of cerebral infarction model (**Table 1**); the remaining eleven animals were used for safety study of intravenous infusion of hMSC and immunosuppressed with Cyclosporine A (**Table 3**). Using the animals in the initial series, we established our MRI protocols (below) and Neurological Scoring protocol (**Table S1**). Five out of eleven animals received hMSC and the remaining six animals received serum from human.

### Anesthetic procedures

Animals were fasted for 12 hours prior to surgery. Anesthesia was induced with Ketamine (10 mg/kg, IM) and Glycopyrrolate (0.015 mg/kg, IM) and maintained via intubation with Isoflurane (1.5–3%) and all efforts were made to minimize suffering. Lactated Ringers (5–10 ml/kg/hour) was intravenously injected during surgery [53].

### Intra-operative monitoring

The animal was placed on a warm water re-circulating heating pad, and covered with a blanket and a second warm water-heating pad. Physiological parameters includes heart rate (HR), mean arterial blood pressure (MABP), body temperature (BT) and oxygen saturation (SaO2) were monitored constantly throughout surgery and recorded every 15 minutes and remained normal before, during and after the cell infusions, and there were no statistically significant differences between groups. (**Tables 2** **,** **4**). Body temperature was measured constantly via a rectal probe.

### MCA occlusion

The animals were fixed to a stereotaxic frame (Kopf, Tujunga, CA) in supine position with their head rotated to the right side. A left frontotemporal pterional approach was carried out. After removal of bone flap, sphenoidal bone was drilled extraduraly. The dura was opened and cut, and the arachnoid and trabecula were dissected out to expose the M1 portion of MCA. A microvascular aneurysm clip (B1 clip, FST, Foster City, CA) was applied to the M1 (**Figure 1A–C**). During the occlusion period, saline soaked gauze pads were applied to the brain surface. The clip was released, dura sealed with fibrin glue and cranioplasty with bone flap and skin closure were performed.

### *Intravenous administration*

After completion of the development series, we performed the safety study of the intravenous infusion of hMSCs into a NHP stroke model. We decided to occlude MCA for 3 hours, release the clip and waited for one hour to close the surgical field and skin. Previous studies using *Macaca* demonstrated that 3 hours of occlusion is sufficient to cause a permanent stroke in the NHP [21], [24]. On the day of infusion, cryopreserved hMSC or human serum were thawed at the surgery room in a 37°C water bath. After a total of four hours, hMSC (1.0×10^7^ cells/18 ml) or human serum (18 ml) was intravenously infused for 30 min using a syringe pump (Harvard Apparatus, Holliston, MA). The Cyclosporin A regimen was 20 mg/kg the day prior to surgery, 15 mg/kg the day of surgery, then 10 mg/kg daily; IM. Cell number was equivalent to the dose that was effective in our previous studies with rodents [6]. The order of delivering hMSC or serum was randomized. The surgeons (M.S and J.D.K) did not know whether hMSC or serum would infuse before and during surgery.

### Post-operative care

The animals were placed on a warm water recirculation-heating pad and covered with a blanket. The animals were monitored a minimum of twice a day for one week or more often as needed. After one week, they were checked at least once a day until stable (eating/drinking normally). Animals unable to feed themselves were hand fed by syringe with a liquid nutritional diet (Bio-Serve, Frenchtown, NJ) until capable of eating themselves and given Lactated ringers subcutaneously.

### Neurological score

Blinded stroke testing using a clinical rating scale (**Table S1**) was performed for the animals in the safety study of the intravenous infusion of hMSCs before and at 1 day, 3 days, 7 days, 14 days, 1 month, 6 months and 12 months after MCAO. The rating scale was modified from a previous study with macaques [29]. Briefly, the state of consciousness, posture, gait with home cage, stimulus and sensation response, extremity and eye movements, as well as hand grasping ability were scored on a point system with a score of zero corresponding to normal behavior and a maximum score of 54 corresponding to severe bilateral neurological impairment.

### Magnetic Resonance Imaging

MRI scans for the animals in the safety study of the intravenous infusion of hMSCs were performed before and at day 3, day 7, month 1, months 5–6, and months 12 post-infarction. The animal was placed in the scanner with the head immobilized. While in the scanner, the animal was maintained on gas anesthesia (isoflurane) and closely monitored. The scanning was performed in a 1.5T MAGNETOM sonata MRI scanner (Siemens Medical Solutions USA, Inc., Malvern, PA, USA). T1- and T2-weighted and Fluid attenuated inversion recovery (FLAIR) images were obtained. The images were obtained in axial and coronal planes. T2-weighted images (T2WI) were obtained from a 3 mm-thick axial and coronal section with 0.9 mm using a 180 mm field of view, TE = 67, TR = 5000 ms, TI = 50 ms, and reconstructed using a 192×256 image matrix. FLAIR were obtained from a 3 mm-thick axial and coronal section with no gap using a 110 mm field of view, TE = 90 ms, TR = 9000 ms, TI = 2200 ms, and reconstructed using a 161×256 image matrix.

The ischemic lesion volume was calculated by a blinded observer from axial plane of FLAIR image using iMac computer (Apple, Cupertino, CA) running OS X and open-source DICOM Viewer software (OsiriX Imaging Software, version 2.7.5, OsiriX Foundation, Geneva, Switzerland). For each slice, the higher intensity lesions in images where the signal intensity was 1.25 times higher than the counterpart in the contra lateral brain lesion were marked as the ischemic lesion area, and infarct volume was calculated taking slice thickness into account [11].

### Histology

The animals were prepared for histological preparation at the end points (**Table 1** **,** **Table 3**). Intracardiac perfusion with saline (2 liters) was followed by 4% paraformaldehyde (1 liter), and the tissue was postfixed overnight [53]. HE staining was performed and to identify the infused hMSCs, immunohistochemisty for anti-human nuclei (dilution 1∶30; Chemicon, Temecula, CA) was carried out on 10 µm frozen sections. Negative controls were performed on stroke-induced monkey brains with human serum infusion.

### *Statistical analysis*

All statistical analysis was performed using SPSS 18 for Macintosh (SPSS, inc; Chicago, IL) and Origin software (version 8.1; OriginLab Corporation, Northampton, MA). Comparison against control group was performed using the Student's t-test. Repeated measures analysis of variance (ANOVA) was conducted for multiple comparisons. Given the small size, we confirmed all results with the Mann-Whitney *U*-test [54].

## Supporting Information

Table S1 **Neurological examination scale.**
(DOC)
