# Supplementary material for: Development of a Middle Cerebral Artery Occlusion Model in the Nonhuman Primate and a Safety Study of I.V. Infusion of Human Mesenchymal Stem Cells
Source: PLoS One. 2011 Oct 24;6(10):e26577. doi: 10.1371/journal.pone.0026577 (PMC3200343; doi:10.1371/journal.pone.0026577)
Supplement: Table S1 — Neurological examination scale. (DOC) [file pone.0026577.s001.doc]

# Table. S1 Neurological examination scale

**1. State of consciousness (0-2):**

0 – normal

1 – drowsy or apathetic

2 – unconscious

**2. Posture- Resting (0-2):**

0 – normal; sitting with head erect

1 – sitting with head hanging

2 – lying down

**3. Gait (0-3):**

0 – normal

1 – limping/ abnormal movement of limb

2 – severely impaired

3 – does not walk, may crawl

**4. Balance (0-2):**

0 – normal

1 – mildly impaired

2 - severely impaired

**5. Posture- During interactions (0-2):**

0 – normal; sitting with head erect

1 – sitting with head hanging

2 – lying down

**6. Visual field test/Tracking Left-Center-Right- Center (0-4):**

Left field – tracks Left 0 – 4 correct

Left field – tracks Right 1 – 3 correct

Right field – tracks Right 2 – 2 correct

Right field – tracks Left 3 – 1 correct

4 – 0 correct

**7. Visual field test/Tracking Right- Center-Left-Center (0-4):**

Left field – tracks Left 0 – 4 correct

Left field – tracks Right 1 – 3 correct

Right field – tracks Right 2 – 2 correct

Right field – tracks Left 3 – 1 correct

4 – 0 correct

**8. Startle-object in from monkey’s right (0-1):**

0 – normal avoidance response/blinking

1 - no response

**9. Startle-object in from monkey’s left (0-1):**

0 – normal avoidance response/blinking

1 – no response

**10. Response to Stimuli right hand (0-3):**

0-moves when barely touched

1-moves when lightly squeezed

2-moves when squeezed hard

3-doesn’t respond

**11. Response to Stimuli right foot (0-3):**

0-moves when barely touched

1-moves when lightly squeezed

2-moves when squeezed hard

3-doesn’t respond

**12. Response to Stimuli left hand (0-3):**

0-moves when barely touched

1-moves when lightly squeezed

2-moves when squeezed hard

3-doesn’t respond

**13. Response to Stimuli left foot (0-3):**

0-moves when barely touched

1-moves when lightly squeezed

2-moves when squeezed hard

3-doesn’t respond

**14. Lip Sensation/Paralysis- Right (0-2):**

0 – normal avoidance response to light touch

1 – responds only to firm tapping on face

2 – doesn’t respond to touch

**15. Lip Sensation/Paralysis- Left (0-2):**

0 – normal avoidance response to light touch

1 – responds only to firm tapping on face

2 – doesn’t respond to touch

**16. Circling (0-1):**

0 – no circling

1 – circling toward the affected side (counterclockwise)

**17. Extremity movement right arm (0-3):**

0–normal

1-assymetrical use or strength

2-minimal movement, profound weakness

3-no voluntary use

**18. Extremity movement right leg (0-3):**

0–normal

1-assymetrical use or strength

2-minimal movement, profound weakness

3-no voluntary use

**19. Extremity movement left arm (0-3):**

0–normal

1-assymetrical use or strength

2-minimal movement, profound weakness

3-no voluntary use

**20. Extremity movement left leg (0-3):**

0–normal

1-assymetrical use or strength

2-minimal movement, profound weakness

3-no voluntary use

**21. Grasping –Right hand (0-2):**

0 – strong grasp; lifts object with hand

1 – weak grasp; wraps fingers around objects

2 – no grasp; doesn’t attempt to use hand

**22. Grasping –Left hand (0-2):**

0 – strong grasp; lifts object with hand

1 – weak grasp; wraps fingers around objects

2 – no grasp; doesn’t attempt to use hand
